# Oxidative Decarboxylation of Levulinic Acid by Cupric Oxides

**DOI:** 10.3390/molecules15117946

**Published:** 2010-11-05

**Authors:** Yan Gong, Lu Lin, Jianbin Shi, Shijie Liu

**Affiliations:** 1 Department of Resources Science and Engineering, State Key Lab of Pulp and Paper Engineering, South China University of Technology, Guangzhou, 510640, China; 2 Department of Paper and Bioprocess Engineering, State University of New York-College of Environmental Science and Forestry, 1 Forestry Drive, Syracuse, NY 13210, USA

**Keywords:** decarboxylation, levulinic acid (LA), 2– butanone, cupric oxides (CuO)

## Abstract

In this paper, cupric oxides was found to effectively oxidize levulinic acid (LA) and lead to the decarboxylation of levulinic acid to 2-butanone. The effects of cupric oxide dosage, reaction time and initial pH value were investigated in batch experiments and a plausible mechanism was proposed. The results showed that LA decarboxylation over cupric oxides at around 300 °C under acidic conditions produced the highest yield of butanone (67.5%). In order to elucidate the catalytic activity of cupric oxides, XRD, AFM, XPS and H_2_-TPR techniques was applied to examine their molecular surfaces and their effects on the reaction process.

## 1. Inroduction

In recent decades, the demand for natural resources has been increasing at an amazing rate, yet fossil fuel sources are on course to be depleted rapidly, while environmental pollution and severe climate changes due to over-exploitation and over-use of fossil fuels gradually become more serious issues. Thus, renewable chemical/energy resources should be urgently developed. Levulinic acid, a short chain fatty acid, is a product of acidic hydrolysis of biomass (such as cellulose and starch) and also one of the extracts from black liquor from paper-making processes [1]. It is a new platform chemical with various potential uses, such as textile dye, antifreeze, animal feed, coating material, solvent, food flavoring agent, pharmaceutical compounds and resin, *etc* [2,3,4].

**Scheme 1:**
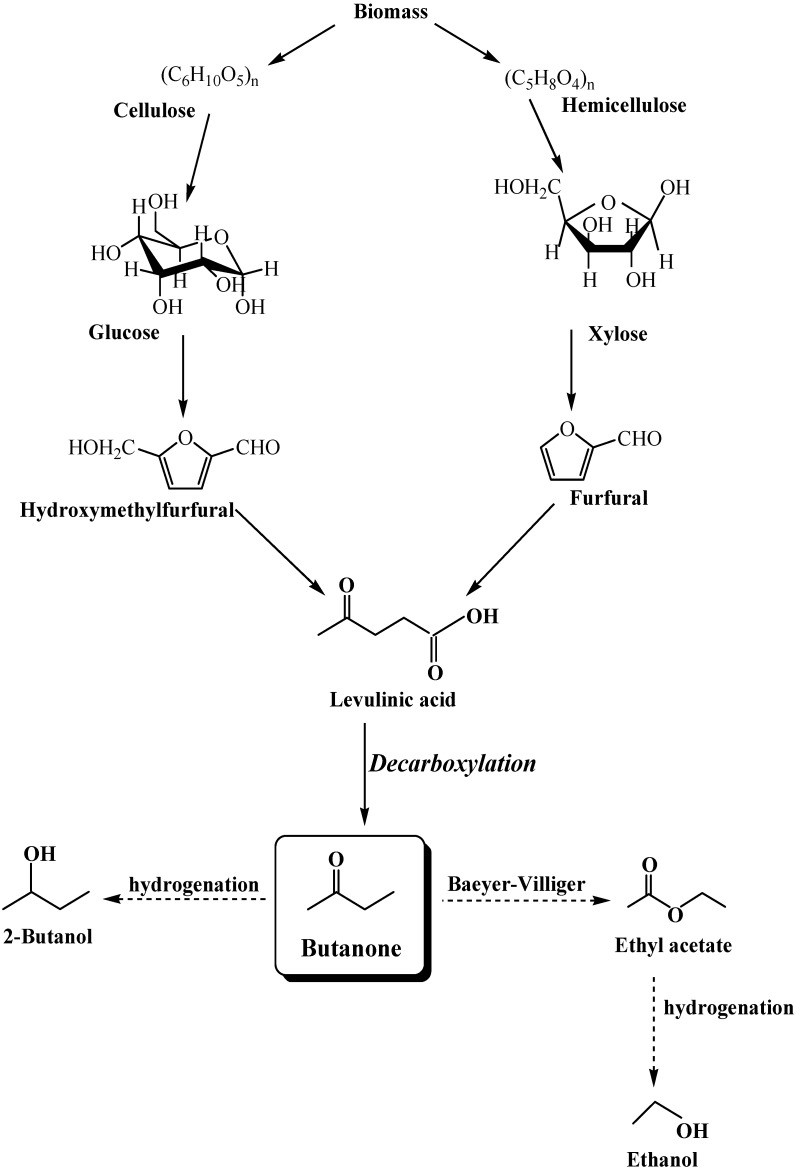
The conversion process of biomass → carbohydrates → fuels.

The reactivity of levulinic acid lies in its molecular structure that contains a carbonyl group and a carboxyl group. Levulinic acid can be hydrogenated to γ-valerolactone [5] and decarboxylated to 2-butanone (methyl ethyl ketone). Butanone is an essential component of the distillate (alcohol oil) derived from the destructive distillation of wood. In industry, it is the product of the dehydrogenation of *sec*-butanol and the aqueous oxidation of butene. It is also produced from methyl formate which reacts with ethylene and carbon monoxide in the presence of a rhodium catalyst with an ionic iodide as promotor, as reported by Mathe [6]. Butanone is usually used as a solvent in organic synthesis, and it can be converted through Baeyer-Villiger oxidation to ethyl acetate [7] which is further reduced to ethanol. Besides, butanone can also be hydrogenated to butanol [8,9], which is a cleaner and superior fuel to ethanol with octane numbers of 113 and 94 as compared with the 111 and 94 of ethanol [10]. Thus, decarboxylation of levulinic acid to produce butanone might be one of the key conversion steps from biomass-derived carbohydrates to versatile fuels (Scheme 1).

Decarboxylation of organic molecules refers to the removal of a carboxyl group from its chemical structure, further replaced by a hydrogen atom. Oxidative decarboxylation of carboxylic acids and their derivatives has been extensively studied over various oxidants involving supported manganese(Ш) porphyrin [11], manganese (III) Schiff base complexes [12], active hydroxyl radicals (•OH) [13], peroxyl radicals [14] and silver(I)/peroxydisulfate [15], *etc*. Thermal decarboxylations of perfluoropolyether salts [16], iron hydroxide cetylsulfonyl acetate [17] and tryptophan [18] have generally received a good deal of attention. Moreover, photolytic decarboxylation (PD) is important in several areas of study and still an area of active research interest. It was commonly used to prepare radical intermediates and organic compounds and also applied in pharmaceuticals and agriculture. Farhadi *et al*. found that α-arylcarboxylic acids were converted into the corresponding aldehydes and ketones selectively by HgF_2_ under photoirradiation conditions in the presence of oxygen gas [19].

To date, efficient and clean methods for decarboxylation of LA have not been previously investigated. Chum [20] explored the photoelectrochemical reaction of levulinic acid on undoped platinized n-TiO_2_ powders, leading to 2-butanone, propionic acid, acetic acid, acetone and acetaldehyde as major products. Even under the most compatible conditions designed, the butanone yield was not satisfactory. In this paper, we report for the first time that LA decarboxylation is catalyzed by CuO and a pathway is proposed for the conversion of LA to butanone.

## 2. Results and Discussion

### 2.1. Surface structures of CuO(I) and CuO(II) with different surface roughness (Rms)

**Figure 1:**
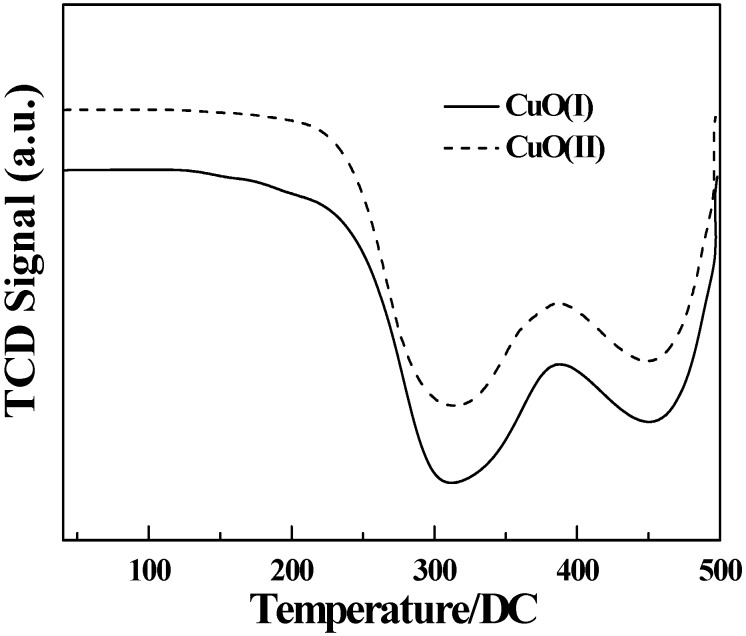
Temperature programmed reduction profiles of CuO(I, II).

CuO(I) was prepared by precipitation of copper nitrate [Cu(NO_3_)_2_] solution with sodium hydroxide (NaOH), CuO(II) was prepared by mixing stoichiometric amounts of oxalic acid and copper acetate in distilled water. The surface roughness (Rms) of CuO(I) and CuO(II) was 123.32 nm and 40.75 nm, respectively. The thermal desorption of hydrogen (H_2_) adsorbed on CuO(I, II) was investigated first to determine their reduction temperatures. A color change from black (CuO) to dark yellow (Cu_2_O) to metallic-brown (Cu) was observed during their reduction process. The TPR profiles are illustrated in Figure 1 and both had two reduction peaks: the first one at about 300 °C and the second one at about 450 °C, indicating the existence of two cupric oxide species. The reduction of isolated Cu^2+^ occurred by a two-step mechanism:

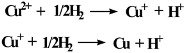



The reduction of CuO took place in a one-step mechanism:

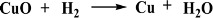


Therefore, the former peak was assigned to the reduction of isolated Cu^2+^ to Cu^+^ and CuO to Cu, while the latter peak was attributed to the subsequent reduction of Cu^+^ to Cu. By comparing the area of two reduction peaks, we observed that the majority of copper was present as isolated Cu^2+^, since the areas of the two peaks were close. It was also indicated that Cu^+^ was more stable than Cu^2+^. The configuration of extra-nuclear electron of Cu was 1s(2)2s(2)2p(6)3s(2)3p(6)4s(1)3d(10). Consequently, the outer electronic configuration of Cu^2+^ and Cu^+^ were 3s(2)3p(6)3d(9) and 3s(2)3p(6)3d(10), respectively. Theoretically, 3d(10) is more stable than 3d(9). Moreover, it was related to the electron attraction ability of the atom core. Atomic power to attract electron in a molecule is relevant to the ratio of effective nuclear charges of ion and atomic radius, that is Z^*^/*r*_i_. The more highly oxidized the atom is, the more positive charge the atom has, the smaller the atomic radius is, the larger Z^*^/*r*_i_ is, and the greater ability to attract electrons the atom has. Thus, Cu^2+^ was more effective in catalyzing the reaction than Cu^+^. When CuO(II) and Cu_2_O were separately used for oxidative decarboxylation of LA at 100 °C for 3 h, the yields of butanone were 0.54% and 0.07%, respectively.

**Figure 2:**
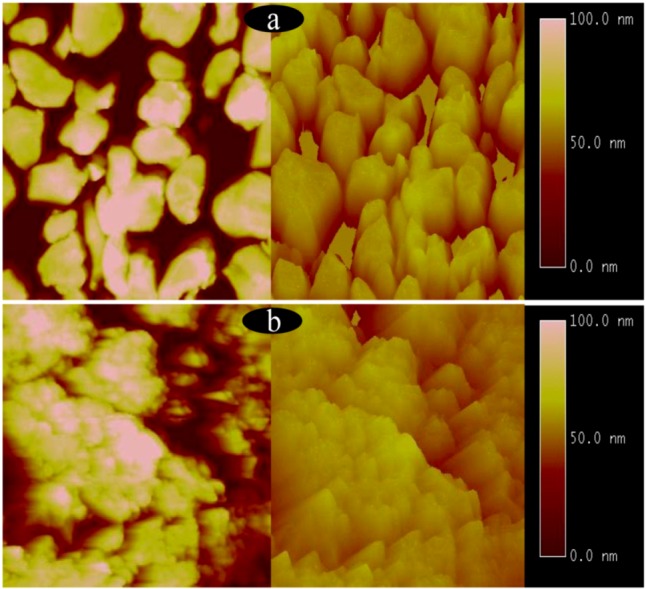
AFM images of two CuO samples [a: CuO(I); b: CuO(II)].

Figure 2 shows the AFM images of two CuO samples which had different surface morphologies. One can observe that the surface of CuO(І) was constituted by uniformly distributed particles (Figure 2a). The surface of CuO(II) seen in Figure 2b was almost entirely composed of agglomerated nanosized grains. The micro-sized agglomerates of nano-sized granules could be regarded as the micro-and nano-scale (namely dual-scale) structure. The surface roughness (Rms) of CuO (I) and CuO (II) was 123.32 nm and 40.75 nm, respectively. This could be attributed to the preparation of CuO (II) that oxalic acid and copper acetate were separately grinded first. 

**Figure 3:**
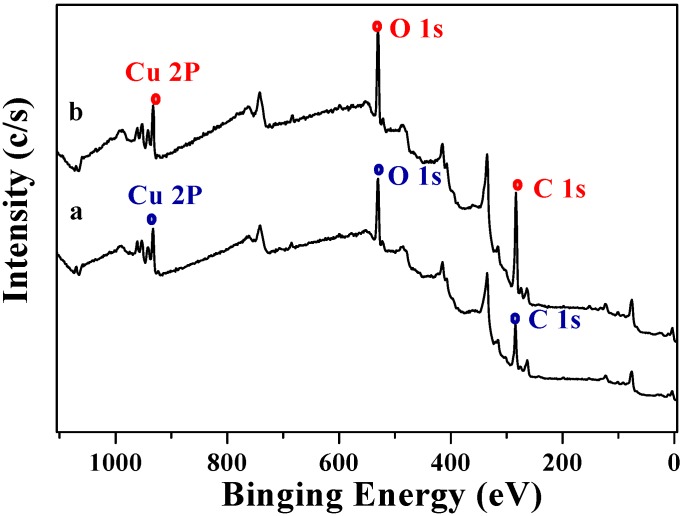
XPS survey spectrum of two CuO samples [a: CuO(I); b: CuO(II)]

**Figure 4:**
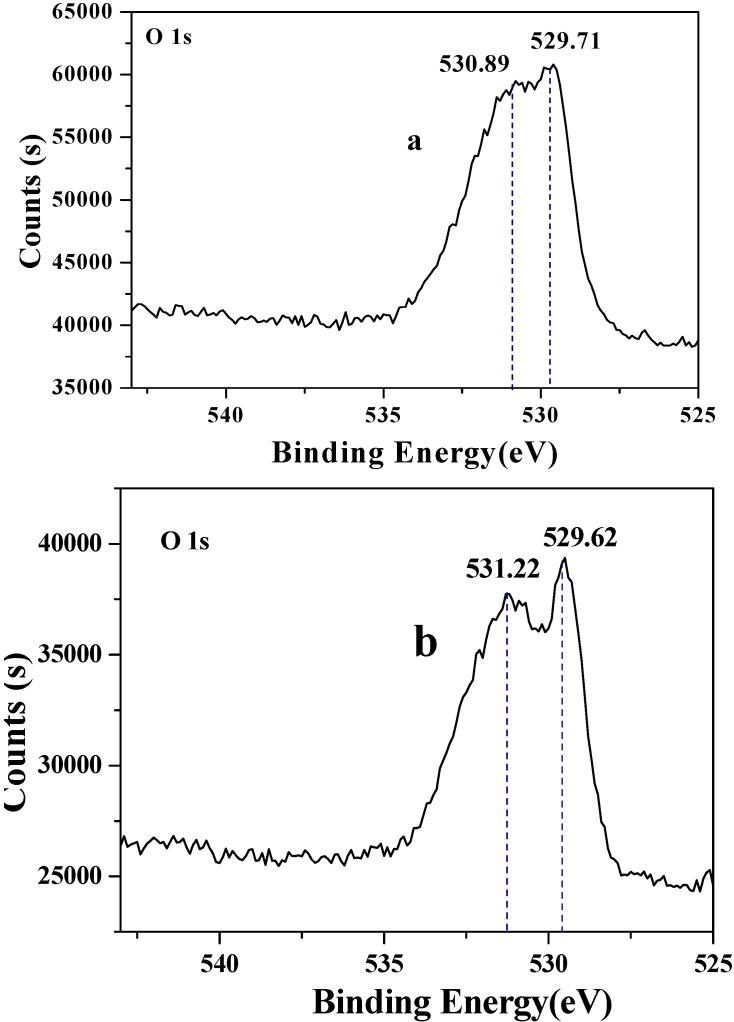
XPS spectra of O 1s peaks of two CuO samples [a: CuO(I); b: CuO(II)].

**Figure 5:**
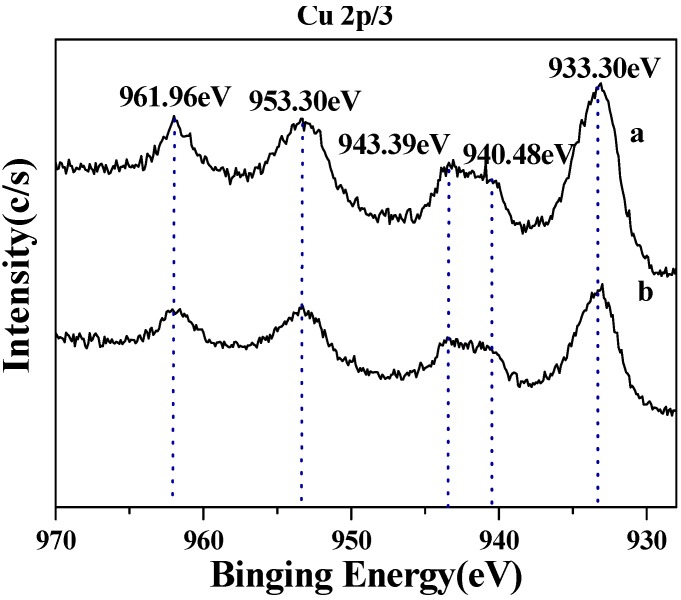
XPS spectra at Cu 2P/3 peaks of two CuO samples [a: CuO(I); b: CuO(II)].

The surface structures of two CuO samples were characterized by XPS and the corresponding survey spectra were shown in Figure 3. All the binding energies were referenced to C 1s peak at 284.6 eV. It could be seen that the surfaces of these two CuO samples were similar. Different elements, essentially carbon, oxygen and copper existed [21,22]. The O 1s core-levels revealed the presence of two components at 529.71 eV and 530.89 eV in Figure 4a and 529.62 eV and 531.22 eV in Figure 4b, both of which corresponded to CuO and Cu(OH)_2_. The peak shape indicated that the Cu 2p/3 spectrum of two CuO samples looked similar (Figure 5). The Cu 2p3/2 core-level peak at 933.30 eV could be assigned to CuO and 940.48–943.39 eV was correlated to Cu(OH)_2_, and the Cu 2p1/2 core-level peak at 953.30 eV and 961.96 eV, respectively.

**Figure 6:**
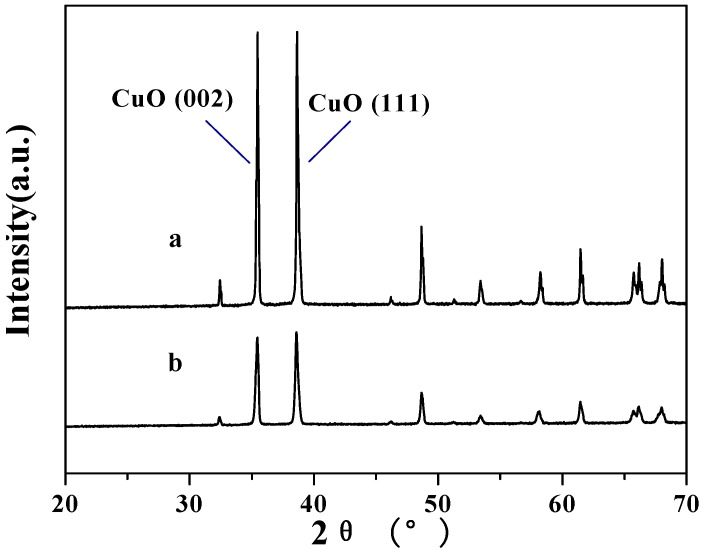
The X-Ray Diffraction spectrum of two CuO samples [a: CuO(I); b: CuO(II)].

The XRD patterns of two CuO samples are shown in Figure 6. The patterns showed two strong peaks at 2*θ* = 35.5° and 38.6° corresponding to (002) and (111) planes of monoclinic CuO, respectively [21,23]. The peaks only corresponding to cupric oxides were observed in Figure 6a and Figure 6b. It showed that single phase CuO was formed in samples of CuO(I) and CuO(II). However, the peak intensity of CuO(І) in Figure 6a was higher than those in Figure 6b, which indicated that the crystallinity of CuO(І) was higher than that of CuO(II).

The grain size of CuO can be roughly estimated by using Scherrer’s formula, D = *kλ/β*cos*θ*, where *β* is the full width half maximum (FWHM) of the strongest diffraction peak, *θ* is the diffraction angle, *λ* is the X-ray wavelength (0.14506 nm), and *k* (~0.89) is Scherrer’s constant. The crystallite sizes of CuO(I) and CuO(II) were 442 nm and 169 nm, respectively. 

### 2.2. Decarboxylation of levulinic acid to butanone catalyzed by CuO(I) and CuO(II)

**Figure 7:**
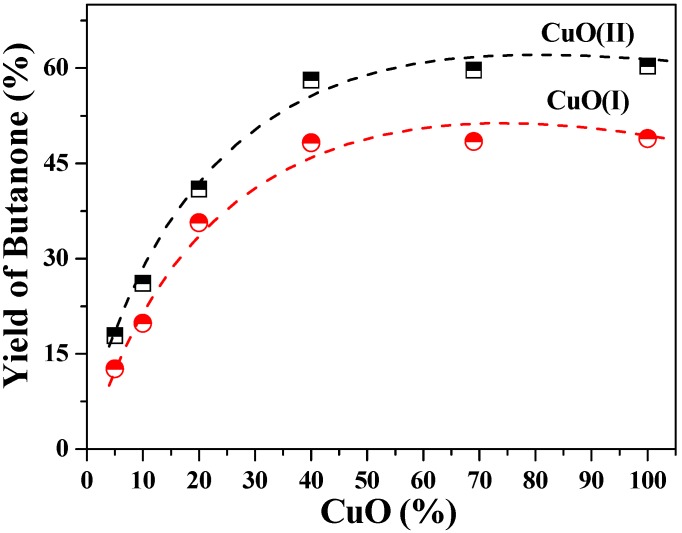
Effect of dosage of two CuO samples on LA decarboxylation.

The LA decarboxylation experiments using these two CuO samples were carried out at 300 °C at pH 7.0. To determine the effect of CuO(I, II) dosages on LA decarboxylation, mass ratios of LA/CuO ranging from 0% to 100% were examined. As seen from Figure 7, increasing both CuO dosages had a positive effect on the reaction that butanone yields increased as the dosage of two CuO samples increased. From 0% to 40%, the yields of butanone increased sharply and reached about 58.2% [CuO(II)] and 48.3% [CuO(I)], respectively. With further increases in CuO dosage, the yields leveled off. We observed that the grain size of CuO(II) was smaller but the butanone yields from decarboxylation of LA by CuO(II) were higher than that by CuO(I). It seemed that the decrease in crystallinity made the CuO(II) more accessible to LA decarboxylation.

**Figure 8:**
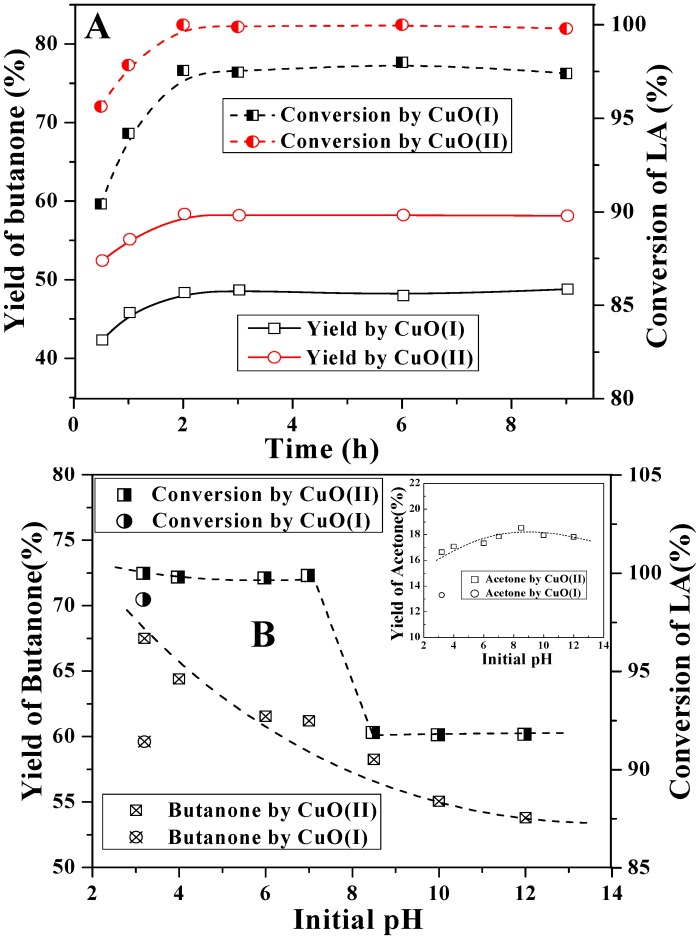
Effect of reaction time (A), initial pH value (B) on LA decarboxylation.

The effect of reaction time from 0.5 h to 9 h on LA decarboxyaltion by CuO(I) and CuO(II) was then investigated. It can be seen from Figure 8A that butanone yields and LA conversions by CuO(II) were higher than that by CuO(I). During first 2 h, LA conversions reached about 97.6% and 100%, respectively. As the reaction time increased to 9 h, no distinct changes were observed. It indicated that the reaction was almost completed within 2 h.

The initial pH value of the reaction solution played a significant role on the reaction. Experiments were carried out at initial pH values ranging from 3.2 to 12. In Figure 8B, at pH ≤ 7.0, LA conversion by CuO(II) was about 100%. With the increase of pH value, it decreased to about 92%. Not much seemed to have changed regarding acetone yields, which fluctuated between 16.7% and 18.5%. CuO(I) was also used as oxidant for LA decarboxylation at pH of 3.2 for a comparison, but butanone yield, acetone yield and LA conversion were all lower than that seen with CuO(II). The pH dependence was similar to that obtained by Chum [20] for the photoelectrochemical reaction of levulinic acid, in which the optimum pH for LA decarboxylation was 4.0. In our experiments, the maximum butanone yield (67.5%) occurred at pH 3.2. It was the lowest pH value as levulinic acid was just mixed with KH_2_PO_4_. According to the results, acidic pH was more favorable to LA decarboxylation than neutral and alkaline pH values. The higher butanone yields at lower pHs were in agreement with the fact that CuO should be more easily dissolved in acidic systems.

**Figure 9:**
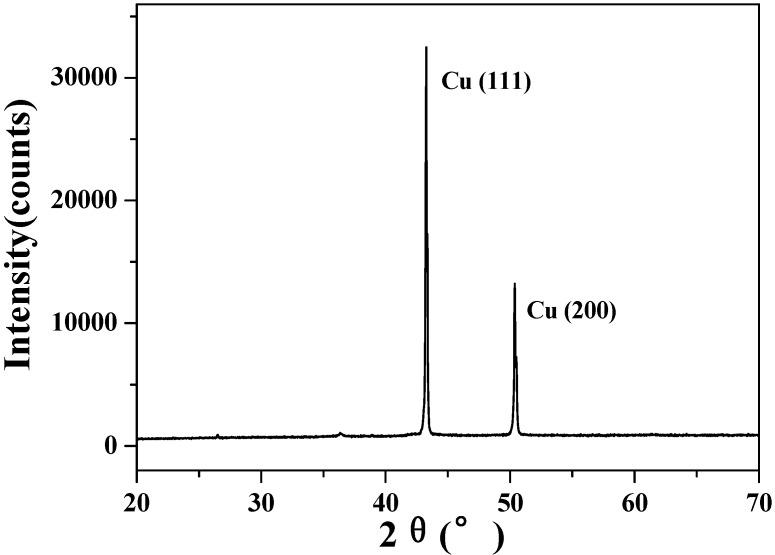
The X-Ray diffraction spectrum of the precipitate after the reactions.

After the experiments, the precipitate was filtered and washed with de-ionized water and then dried in an oven to give a reddish solid that was subsequently examined by XRD. As seen in Figure 9, the peaks corresponding to CuO(II) in Figure 6 disappeared and peaks corresponding to Cu appeared instead [24]. Thus, we can conclude that CuO was completely reduced to Cu at 300 °C.

Based on the TPR profiles of CuO(I, II), the precipitate after the experiments should be a mixture of Cu and Cu_2_O (in a high proportion). But Figure 9 only showed the peaks corresponding to Cu. Thus, how Cu_2_O changed into Cu in this reaction needs further study. Besides, the temperature of 300 °C seemed extreme. The search for milder reactive conditions together with high catalytic efficiency is imperative for potential industrial applications and a meaningful organic synthesis method. It was reported that TPR peak temperatures of CuO/CeO_2_ catalyst [25], CuO/Al_2_O_3_ catalysts [26] and copper-exchanged mordenites (CuMOR) [27], *etc.* were much lower than that of pure CuO. One can extrapolate that LA may be decarboxylated at lower temperatures using these CuO-modified catalysts and this is under study.

### 2.3. Reaction pathway for LA decarboxylation by CuO

**Figure 10:**
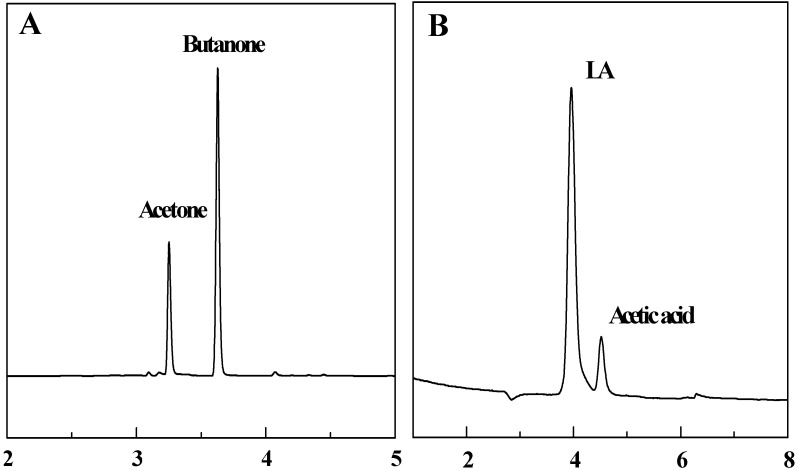
Head space - gas chromatogram (A) and Ion Chromatogram (B) of samples.

**Scheme 2:**
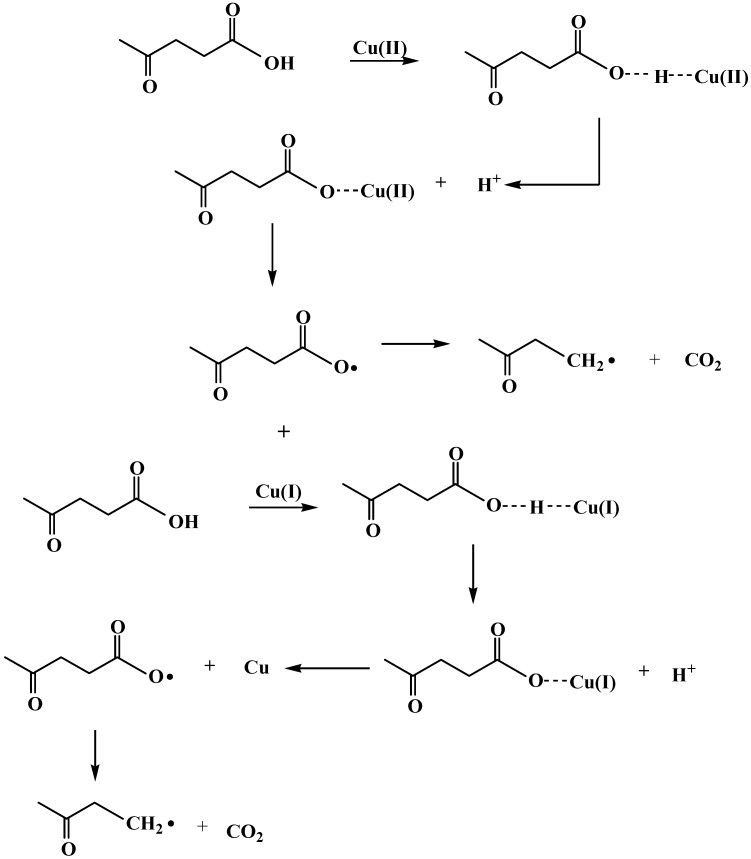
The proposed reaction pathway for LA decarboxylation by CuO samples.


According to the experimental analysis by HS-GC-MS shown in Figure 10A and by ion chromatography in Figure 10B, LA could be oxidatively decarboxylated to butanone by CuO(I, II) and heat treatment. The oxidative decarboxylation of LA is given by eq. 1. From the butanone yields and LA conversions at different Initial pH values as shown in Figure 8B, we may deduce that the mechanism of LA decarboxylation in acid solution is different from that in alkaline solution. Many organic compounds, such as ethyl stearate and *ortho*-substituted benzoic acids, *etc.* [28,29,30] are decarboxylated in free radical reactions. Our observations showed that butanone yields and LA conversions were higher under acidic conditions. In other words, the free radical reaction was much more compatible with LA decarboxylation under acidic pH. A plausible pathway as shown in Scheme 2 is proposed to explain the experimental observations. We propose that it is the Cu(II) species which is the agent directly responsible for LA decarboxylation. The initial attack of Cu(II) is by addition of LA producing hydrogen bond, then the hydrogen atom transfers to a free hydrogen ion and meanwhile a Cu-oxygen bond is formed. The oxidation may occur via prior incorporation of LA into the Cu(II) species as a ligand followed by homolysis of the Cu-O bond. Then the acyloxy acyl radical (CH_3_COCH_2_CH_2_CO_2_•) is known to undergo a rapid fragmentation with liberation of carbon dioxide (CO_2_) and an alkyl radical (CH_3_COCH_2_CH_2_•). Butanone (CH_3_COCH_2_CH_3_) is then derived by hydrogen atom transfer to solvent (eq. 2). Cu)(II) was firstly reduced to Cu(I) followed in succession by a second reduction to the elemental form (Cu):

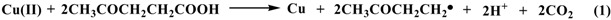


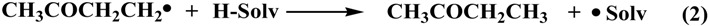



When the experiments were carried out in alkaline conditions, the covalent bond may be cleaved by a heterolysis process that is a two-step ionic decarboxylation as indicated in eq. 3. Levulinic acid is first dissociated into a carboxylic anion (CH_3_COCH_2_CH_2_CO_2_^−^) which then undergoes a heterolytic cleavage of the alkyl-carbonyl bond and leads to the formation of an alkyl anion (CH_3_COCH_2_CH_2_^−^) and CO_2_. The alkyl anion has strong basicity and it can combine with hydrogen ion to become 2-butanone [31,32]. Acetic acid and acetone may be produced by breaking the C-C bond between the α-carbon and β-carbon because of the electron attraction effect of carboxyl and carbonyl group.

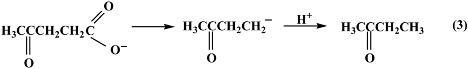


## 3. Experimental

### 3.1. Preparation of CuO samples

CuO(I) was prepared by precipitating copper nitrate [Cu(NO_3_)_2_] solution with sodium hydroxide (NaOH). The precipitate was washed with deionized water and then dried in a vacuum oven at 50 °C for 4 h. The dried sample was calcined in a muffle furnace at 800 °C for 2 h. CuO(II) was prepared by mixing stoichiometric amounts of oxalic acid and copper acetate in distilled water. The resultant blue solid (CuC_2_O_4_) was washed with deionized water and then dried in a vacuum oven at 70 °C for 4 h. The prepared sample was ground and subsequently calcined in a muffle furnace at 800 °C for 2 h. CuO(I) and CuO(II) were used in LA decarboxylation and further analyses.

### 3.2. Procedure for LA decarboxylation

All experiments were carried out in a 4576-HP/HT Pressure Reactor (200 mL, Parr Instruments). Reaction solutions were obtained by adding levulinic acid (0.01 mol) into KH_2_PO_4_-NaOH (0.2 mol/L) solution and the initial pH of the reaction solution was adjusted with NaOH (0.2 mol/L). The total volume was 60 mL. Then a metal oxide or metal-oxides mixture was added into the prepared reaction solutions. After the reactions were completed, the samples were taken with 5 mL pipette and directly transferred to a 20 mL headspace vial for GS-GC detection. In view of the volatility of products, three samples were taken and the average value of the data was recorded. And the samples were filtered through a 0.25 μm membrane filter for ion chromatography detection.

### 3.3. Head space – gas chromatography detection

Head space (HSS 86.50)-gas chromatography (QP2010, Shimadzu Japan) (HS-GC) was used extensively in the separation and identification of mixtures of volatile compounds. LA decarboxylation samples were analyzed with a HS-GC using a DB-5 column (30 m × 0.25 mm i.d. × 0.25 μm film) at a column temperature of 120 °C, a flow rate of 4.7 mL/min and with a flame ionization detector at the temperature of 220 °C. The analysis was performed at 60 °C for 5-min. The component concentrations were calculated by the area normalization method. The sample vials was shaken for 20 min at 75 °C. The temperatures of sampling probe and tube were 85 °C and 95 °C, respectively. Automatic sampling was employed. The sample analysis was confirmed by the head space-gas chromatograph-mass spectrometer technique (HS-GC-MS). Some samples were detected with a HS-GC-MS (Agilent 6890-5975) equipped with a DB-5 column (30 m × 0.25 mm i.d. × 0.25 μm film). The temperature program was used: 60 °C –(5 min) −10 °C/min −120 °C. The carrier gas was helium with a flow rate of 1.0 mL/min and the mass spectrometer used an electron impact (EI) ionization mode with 70 eV of electron energy. The HS-GC of samples shown in Figure 10A confirms that acetone and butanone were the gas products and the retention times were about 3.26 min and 3.63 min, respectively.

### 3.4. Ion chromatography detection

Ion chromatography (ICS-3000, Dionex USA) was used for the analysis of levulinic acid in the aqueous phase to evaluate the conversion of levulinic acid. Liquid reactant after the reactions was filtered and diluted before testing. The analysis was conducted in an anion-exchange column (AS11-HC) with 2 mol/L NaOH leacheate in a conductivity cell of 35 °C. The flow rate was 1.0 mL/min and the sample size was 50 μL. The calibration was performed using aqueous solutions. An ion chromatogram of a sample is shown in Figure 10B, where the LA peak was between 3.95 min and 4.06 min.

### 3.5. X-ray diffraction analysis

The grain size of CuO powders was characterized by X-ray diffraction (XRD) (D/max-IIIA, Rigaku, Japan) (40 kV, 40 mA) using the standard reflection mode with Cu/Ka (λ = 0.154056 nm) in the angle 20° < 2θ < 70° and step angle was 0.02°.

### 3.6. Atomic force microscope measurement

The surface morphologies of CuO powders were observed by atomic force microscope (AFM) (Nanoscope IIIa Scanning, Digital Instruments, USA) using tapping mode with an RTESP cantilever.

### 3.7. X-ray photoelectron spectrometry

To determine the surface compositions of CuO, X-ray photoelectron spectroscopy (XPS) was carried out with an Axis Ultra (DLD) spectrometer (Kratos, 5 mA, 15 KV) using Al Kα source and Mg Kα source. Binding energy was calibrated with hydrocarbon- contaminated C1s peak at 284.6 eV at ambient temperature.

### 3.8. Temperature programmed reduction analysis

The temperature programmed reduction (TPR) measurement of CuO powders was carried out using an AutoChem II 2920 Chemisorb system from room temperature to 500 °C at a heating rate of 10 °C/min by flowing 50 mL/min of H_2_ (10 vol.%)/Ar. The amount of H_2_ uptake during the reduction was measured by a thermal conductivity detector (TCD). The water produced during the reduction was trapped in a 5Å molecular sieve column.

## 4. Conclusions

It is shown based on above discussions that butanone can be produced through oxidative decarboxylation of biomass–derived levulinic acid with CuO. CuO itself was reduced to its elemental form (Cu). The LA decarboxylation is the result of a combination of CuO’s oxidability, structure (transition metal with unoccupied orbital) and external conditions (temperature, pH, *etc*). An acidic system helped to facilitate the oxidative decarboxylation of LA. At pH 3.2, the butanone yield reaches 67.5%.
